# Localized States’ Role in MoS_2_ Few-Layer Device: A Study by Schottky Capacitance Spectroscopy and Thermally Stimulated Current

**DOI:** 10.1021/acsomega.6c06145

**Published:** 2026-08-12

**Authors:** Riama Coelho Gouveia, Leonélio Cichetto, Luciano Ferreira de Almeida, Marcio Daldin Teodoro, Adenilson José Chiquito

**Affiliations:** † NanOLaB, Universidade Federal de São Carlos, Rod. Washington Luís, Km 235 - SP 310, São Carlos, São Paulo 13565-905, Brazil; ‡ Instituto Federal de Educação Ciência e Tecnologia de São Paulo, Campus Sertãozinho, Rua Américo Ambrósio, 269, Sertãozinho, São Paulo 14169-263, Brazil; § Instituto Militar de Engenharia, Praça Gen. Tibúrcio, 80, Rio de Janeiro, Rio de Janeiro 22290-270, Brazil; ∥ Departamento de Física 67828 Universidade Federal de São Carlos, Rod. Washington Luís, Km 235 - SP 310, São Carlos, São Paulo CEP 13565-905, Brazil

## Abstract

This paper reports on the Chemical Vapor Deposition synthesis of few-layer molybdenum disulfide (MoS_2_) films, with their two-dimensional nature confirmed by Raman and photoluminescence spectroscopies. Raman analysis revealed a frequency separation of 22.2 cm^–1^ between the $E_{2g}^{1}$ and A_1g_ modes, indicating a structure with fewer than 4 atomic layers. To investigate interface and transport properties, planar diode-type devices were fabricated using Au and In/Au contacts. Temperature-dependent electrical characterizations demonstrated that charge transport is governed by a 2D Variable Range Hopping mechanism, highlighting the influence of localized states. Through Thermally Stimulated Current (TSC) spectroscopy, we identified trap levels with an activation energy of approximately 120 meV, attributed to sulfur vacancies. Additionally, Schottky Capacitance Spectroscopy (SCS) revealed a nearly uniform distribution of interface states with a density on the order of 10^11^ cm^–2^ eV^–1^. The high consistency between TSC and SCS results confirms that localized states dominate both the metal–semiconductor contact behavior and the overall charge transport in few-layer MoS_2_ devices.

## Graphical abstract

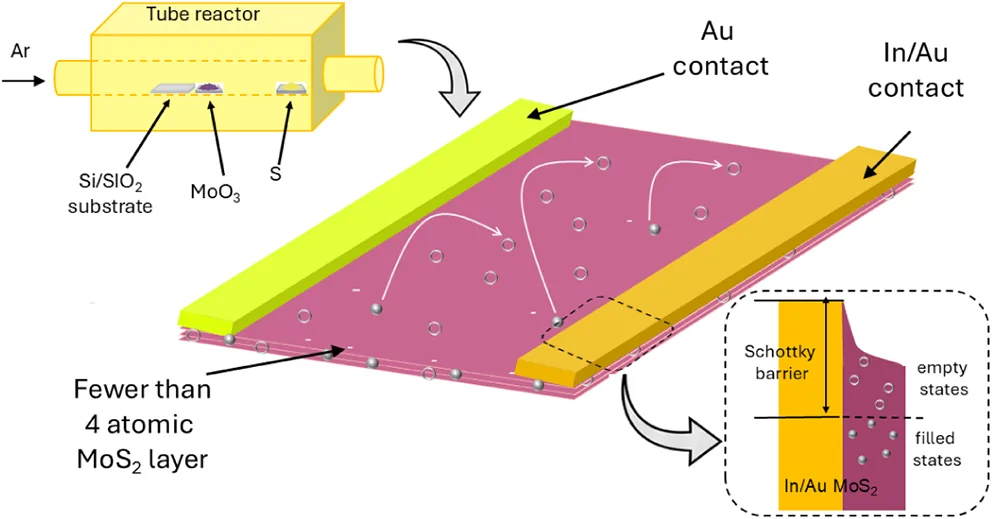

## 1 Introduction

Two-dimensional (2D) transition metal dichalcogenides (TMDCs) are highly promising materials for both fundamental studies and ultrathin device technology with applications in logic circuits, energy storage, flexible electronics, optoelectronic devices, and sensors.^1–7^ The appeal of these 2D materials lies in their strong light-matter interaction and tunable bandgap with strong photoluminescence, layer–layer interactions via Van der Waals forces, surface effects due to large surface-to-volume ratio, strong spin–orbit coupling, large exciton binding energy, quantum spin Hall effects, and valley polarization.^1,4,5,8^ Monolayer molybdenum disulfide (MoS_2_), in particular, turns from an indirect gap into a direct band gap in the visible range (∼1.9 eV) with strong emission, presents a high on–off current ratio that requires low standby power, and has good electrical performance, making it a promising material for electronic circuits, photocatalysis, and optoelectronics applications.^2,9,10^

The preparation of few-layer MoS_2_ can be carried out using different techniques (methods), such as scotch tape-based micromechanical exfoliation, intercalation-assisted exfoliation, liquid exfoliation, hydrothermal synthesis, thermolysis of a single precursor containing Mo and S, sulfurization of precursor films, physical vapor deposition (like pulsed laser deposition), and chemical vapor deposition (CVD).^3,9,11^ Among these approaches, CVD synthesis is the simplest and most cost-effective, allowing for the growth of large areas of high-quality single-crystalline 2D MoS_2_.

Most characterizations and applications involve the construction of devices and, therefore, the establishment of electrical contacts between metals and MoS_2_. The behavior of the contact—ohmic or rectifying—and other contact properties like contact resistance are related to the physics of the interface between the metal and the semiconductor.^12–16^ Some previous research indicates that localized energy states at the metal/2D MoS_2_ interface play a significant role in both contact behavior and charge transport of 2D MoS_2_ devices,^17,18^ as illustrated in the band diagrams in Figure 1.

**Figure 1:**
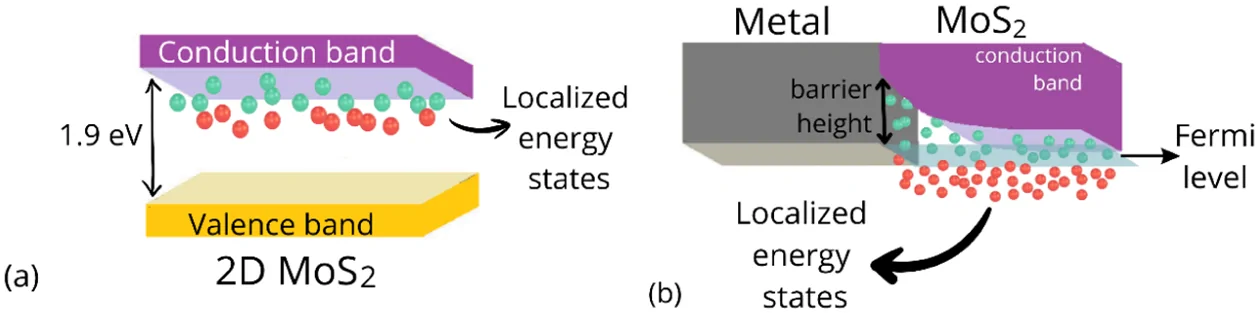
Schematic band diagrams: (a) conduction band, valence band, and the bandgap of few-layer MoS_2_, with the presence of localized energy states; (b) localized energy states in MoS_2_-metal interface.

Bampoulis et al. demonstrate that subsurface metal-like defects (with a density of ∼10^11^ cm^–2^) induce strong Fermi level pinning, drastically reducing the Schottky barrier height compared to pristine regions.^17^ While the pinning factor in pristine areas is ∼0.3, it drops to ∼0.1 at defect locations, creating low-resistance conduction paths that become dominant as the device junction contact area decreases in size.^17^ Complementarily, McDonnell et al. observed that intrinsic defects dominate the doping and resistance of MoS_2_, providing low Schottky barriers that are largely independent of the metal work function.^18^ Furthermore, the interface chemistry is highly sensitive to fabrication conditions.^12^ Interfacial reactions can transform “van der Waals” interfaces into covalent ones depending on the metal’s reactivity (such as Cr and Sc) and the deposition environment (ultra-high vacuum vs high vacuum), which significantly alters the conduction barrier.^12^

In high-performance devices, such as dual-gated bilayers encapsulated in hexagonal boron nitride (h-BN), transport understanding extends into the quantum regime.^19^ Qu et al. identified the weak localization effect at low magnetic fields (|B| < 0.5 T), allowing for the extraction of fundamental parameters like the phase coherence length. At electron densities exceeding 7 × 10^12^ cm^–2^, coherence length can exceed 100 nm. Their results suggest that Coulomb interaction is the primary dephasing mechanism in the single-band regime. Notably, the absence of weak antilocalization indicates that the intrinsic spin–orbit coupling of MoS_2_ protects spin states through spin-valley locking, resulting in spin relaxation lengths much longer than the phase coherence length.

Despite these advances, contact phenomena and the mutual influence between defects and quantum properties are not yet fully understood.^19,20^ Therefore, a detailed investigation into the distribution, density, and chemical nature of these localized states, along with electronic scattering mechanisms, is essential for engineering interfaces that minimize resistance and enable the precise control of carrier populations across different spin bands.

In this paper, we report the CVD synthesis of few-layer MoS_2_ films, confirming their two-dimensional nature through Raman spectroscopy (noting a characteristic peak separation of 22.2 cm^–1^) and photoluminescence (strong emission at approximately 1.9 eV). Employing Schottky Capacitance Spectroscopy (SCS) and Thermally Stimulated Current (TSC) measurements in planar diode-type devices, we identified a nearly uniform distribution of interface states with a density on the order of 10^11^ cm^–2^ eV^–1^ and trap levels with an activation energy around 120 meV. We demonstrate how these localized states, attributed to sulfur vacancies, govern charge transport via a 2D Variable Range Hopping (VRH) mechanism and dictate the temperature dependence of the Schottky barrier height, providing a better understanding of the interfaces between gold/indium and MoS_2_, as well as the surface physics in two-dimensional MoS_2_ materials.

## 2 Experimental Approach, Results, and Discussion

### 2.1 Synthesis and Device Fabrication

Usually, in the CVD process, powders of metal oxides and chalcogens used as precursors are placed inside a tube reactor and heated to appropriate temperatures to be evaporated in the presence of an inert carrier gas that transports them to the substrates, where they are thermally activated to react and form 2D structures.^3^

In our synthesis, approximately 50 mg of molybdenum oxide (99.5%, ACS Reagent) and 500 mg of sulfur (99.5%, Sigma-Aldrich), the precursor powders, were placed in a tube reactor (Lindberg/Mini Mite) at positions corresponding to temperatures suitable for the evaporation of each material: MoO_3_ at the center of the reactor, at 850 °C, and sulfur at a temperature, during the synthesis period, of approximately 200 °C. A silicon substrate (Silicon Sense, n-type) with a thermal silicon oxide layer (500 nm) previously cleaned was placed at the center of the reactor and used as the growth substrate. The reactor was heated to 850 °C and held at that temperature for 5 min; simultaneously, the pressure was reduced to approximately 15 kPa while a flux of 15 sccm of argon (Air Liquide, >99.998%) was used to carry the evaporated precursors to the substrate. After this time, the reactor was turned off and naturally cooled to room temperature.

The substrates removed from the synthesis reactor were then examined under an optical metallographic microscope (Zeiss Axio M2m). The observation under a microscope revealed that the substrate was partially covered by a thin film of MoS_2_, indicating that the synthesis process was successful, as confirmed by Raman and photoluminescence (PL) spectroscopies, which were used to confirm the growth of the intended structure and its 2D nature.

Raman spectra were taken at room temperature and pressure using a homemade confocal setup equipped with a diode laser operating at 532 nm (Cobolt-08) focused on a ∼1 μm spot. The Raman signal was dispersed by a 75 cm spectrometer with a grating of 1200 l/mm and detected by a silicon charged-coupled device (Andor/Shamrock—iDus); the Si peak at 520.7 cm^–1^ was used for peak position calibration.

Figure 2a displays the Raman spectrum of the one spot of the synthesized sample, exhibiting the characteristic $E_{2g}^{1}$ (in-plane) and A_1g_ (out-of-plane) vibrational modes located at 384.4 cm^–1^ and 406.6 cm^–1^, respectively. The other two spectra obtained from different locations present similar results. The data were fitted with Lorentzian functions, revealing a peak frequency separation of 22.2 cm^–1^. Compared to bulk values (∼383 cm^–1^ for $E_{2g}^{1}$ and 408 cm^–1^ for A_1g_), a clear red-shift of the $E_{2g}^{1}$ mode and a blue-shift of the A_1g_ mode are observed. This spectral evolution, governed by the interlayer van der Waals coupling and the screening of long-range Coulombic interactions, serves as an unambiguous signature of few-layer structures, confirming that the synthesized film consists of fewer than 4 atomic layers.^21,22^

**Figure 2:**
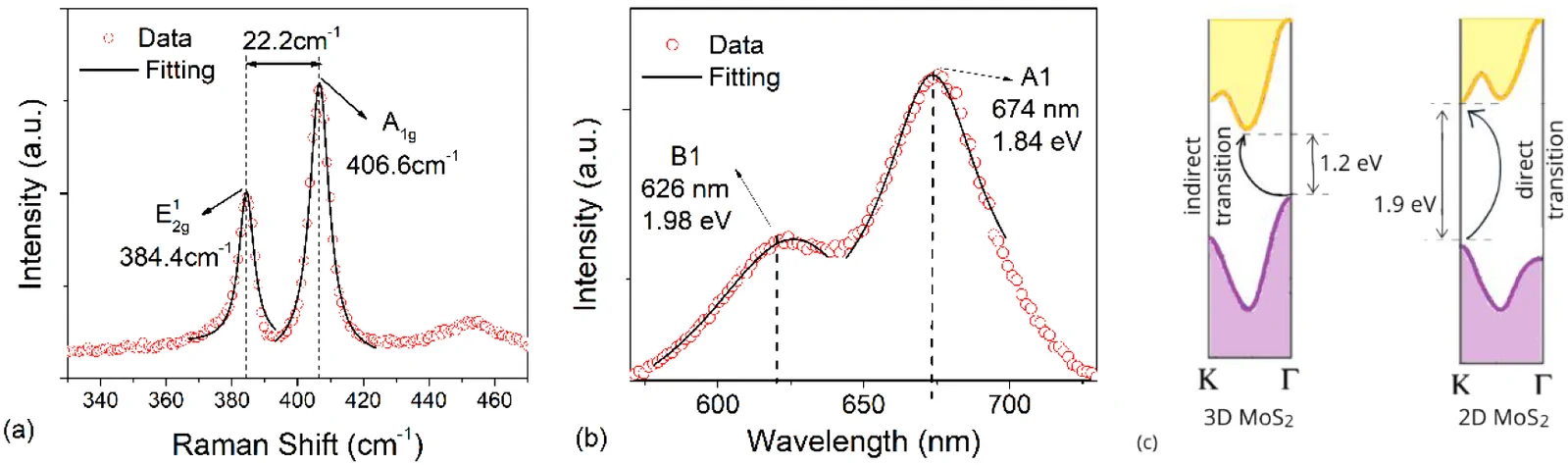
(a) Room temperature Raman spectrum from a few-layer MoS_2_ sample. Black lines are Lorentzian fitting curves. Vertical dashed lines indicate the peak positions. (b) Room temperature PL spectrum of a few-layer MoS_2_ film. Experimental points are presented as red circles, and fitting results are given by the solid black line. The positions of B1 and A1 peaks, related to direct excitonic transition energies, are marked with a dashed vertical line; (c) schematic band diagram showing photoluminescent transitions on 2D and 3D MoS_2_.

PL spectra from four different locations of the sample were taken using the same Raman setup, but the signal was dispersed and detected by a 30 cm spectrometer and a silicon CCD (Andor/Kymera—iDus). All PL spectra obtained were similar, and the spectrum in Figure 2b, presented as an example, clearly shows two emission peaks around 625 and 675 nm that are related to B1 and A1 direct excitonic transitions, respectively. As illustrated in Figure 1c, the optical bandgap of MoS_2_ changes from indirect (∼1.2 eV) to direct (∼1.9 eV) when the dimension is reduced from a bulk form to a monolayer sheet, which increases the emission intensity of direct transitions with the decrease in the layer number.^23,24^ Since the direct excitonic transition luminescence is absent in the indirect bandgap bulk MoS_2_, the pronounced peak obtained in the PL spectra agrees with Raman results, confirming that the studied sample is indeed a few-layer structure.

Once the presence of a few-layer MoS_2_ film on the substrates was verified, devices in a metal–semiconductor-metal (MSM) architecture were fabricated using a direct-write photolithography system (Durham ML3 Baby Microwriter). Figure 3 depicts a schematic representation and an optical image of a device. Thermal evaporation (Edwards AUTO 306) under high vacuum (better than 10^–6^ mbar) was used to deposit metal contacts.

**Figure 3:**
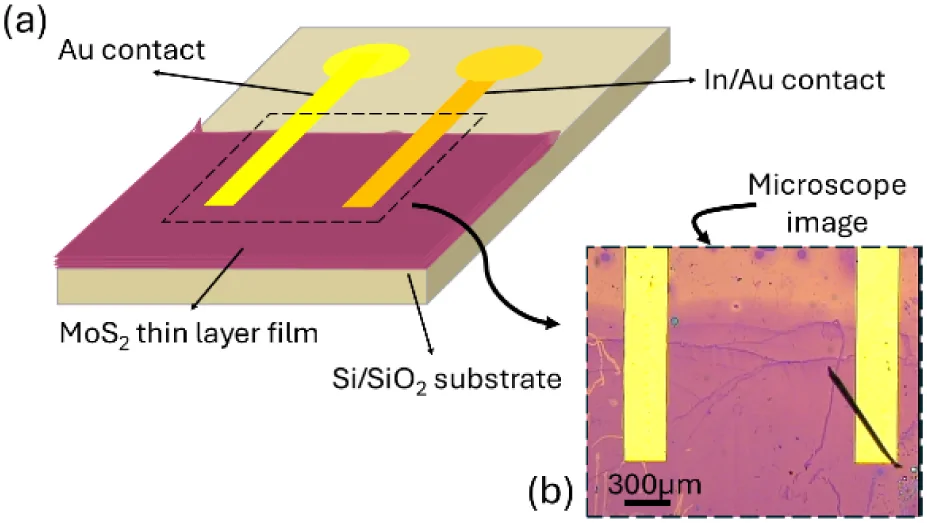
(a) Schematic of the few-layer MoS_2_ diode-type device and (b) an optical image obtained using a metallographic microscope (Zeiss Axio M2m) of the actual device.

Considering that a diode-type device allows investigation of both charge transport in the semiconductor and the characteristics of the interface between the semiconductor and the electrode metal, different metals were used in the construction of the device, allowing the production of an Ohmic contact on one side and a rectifier contact on the other. As literature reports the Au-MoS_2_ interface as exhibiting ohmic behavior,^25^ gold (80 nm thick) was chosen for one of the contacts. Indium was the choice for the rectifier contact, additionally coated with a layer of gold to prevent oxidation (In/Au, 20/80 nm thick).

When selecting metals for the contacts, we also considered that some metals react with MoS_2_ to form interfacial reaction products that add chemical states. In such cases, the study of carrier transport becomes more complex, making it difficult to obtain data on MoS_2_. For example, when metallic titanium is used to define the contacts, it may form TiO_2_ or react with MoS_2_, forming Ti_*x*_S_*y*_ and metallic Mo at the interface, depending on the deposition conditions.^26^ Therefore, gold was chosen based on research by Smyth et al.,^12^ which affirms that the evaporation of this metal in high vacuum is not expected to react or create additional states in the MoS_2_/metal interface. Similarly, and based on studies by Rahman et al., indium was selected because it does not damage the MoS_2_, thereby avoiding the generation of new states.^14^

### 2.2 Electrical Characterization

Current–voltage (I–V) characterization at different temperatures (200–300 K) was carried out using a high voltage source-measure unit (Keithley 237) and a closed-cycle helium cryostat (Janis CCS 400H) working at a pressure lower than 10^–5^ mbar. In Figure 4a, we observe the device’s diode-like behavior in the whole temperature range.

**Figure 4:**
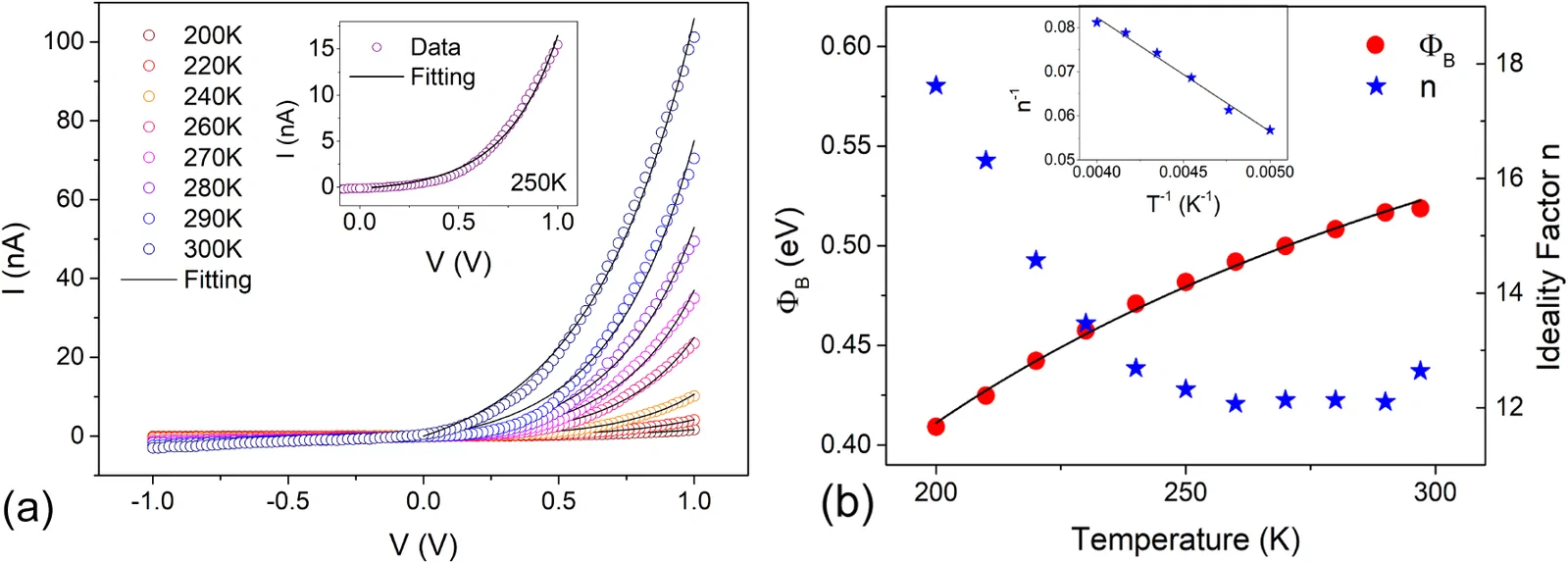
(a) Current–voltage curves taken at different temperatures. The inset illustrates the thermionic emission fitting of the curve taken at 250 K. Temperature-dependent Schottky barrier height (Φ_*B*_) and ideality factor (*n*) are depicted in panel (b). The inset shows the ideality factor according to the Gaussian distribution of localized energy states.

According to the thermionic emission, the current through the Schottky barrier is given by eq 1:^27^

1 $$I\left(V\right)=SA^{**}T^{2}\exp\left(\frac{-e\Phi_{B}}{kT}\right)\exp\left[\left(\frac{eV}{nkT}\right)-1\right]$$

where *S* denotes the area, which is effective for current transport, A** is the effective Richardson constant, Φ_*B*_ is the Schottky barrier height, *n* is an empirical ideality factor, and the other symbols have their usual meanings.

The barrier height energies obtained by fitting the I–V curves using eq 1 are presented in Figure 4b: there is a clear increase of barrier height with temperature, from 0.42 to 0.52 eV. Despite the Schottky–Mott model, which assumes both the ideality factor and the barrier height should be temperature independent, a variation in the homogeneity of the Schottky contact leading to a distribution of barrier heights was observed by several authors.^27^ In this case, as the temperature increases, the electrons have sufficient energy to overcome higher barriers, and, as a result, the barrier height appears to increase with temperature rises.

Ideality factor values obtained from the same fitting procedure are all larger than one, and while the barrier height increased with temperature, the ideality factor decreased as the temperature rose (Figure 4b). The linear fit of n^–1^/T^–1^, presented in the inset of Figure 4b, is in accordance with the model of a continuous barrier distribution at the interface in which the ideality factor represents the voltage deformation of the barrier distribution.^28^

Values greater than one for ideality factors can be explained by a thin oxide between the metal and the semiconductor, tunneling currents in highly doped semiconductors, or image force lowering of the Schottky barrier in a large electric field.^28^ None of these cases can be applied to the device under consideration, since neither an oxide layer was added nor does MoS_2_ oxidize easily. The selected metals, as mentioned above, do not react with or dope the MoS_2_, and during the measurements, the device was subjected to a small electric field. On the other hand, ideality factors greater than unity in Schottky contacts without an oxide layer between the metal and semiconductor in a nondoped semiconductor may be attributed to local spatial inhomogeneities at the interface between the metal and semiconductor, leading to localized energy states situated in the semiconductor band gap and physically located between the two materials, known as interface states.^28^

The temperature-dependent resistance curves of the device were also obtained. The data shown in Figure 5a revealed the semiconductor behavior of the device, with a monotonic exponential decrease in resistance as the temperature increased in the whole range used for measurements. A more detailed investigation of carrier transport, presented in Figure 5b, shows that the best fit for the data was obtained when using the variable range hopping (VRH) process in a 2D system.

**Figure 5:**
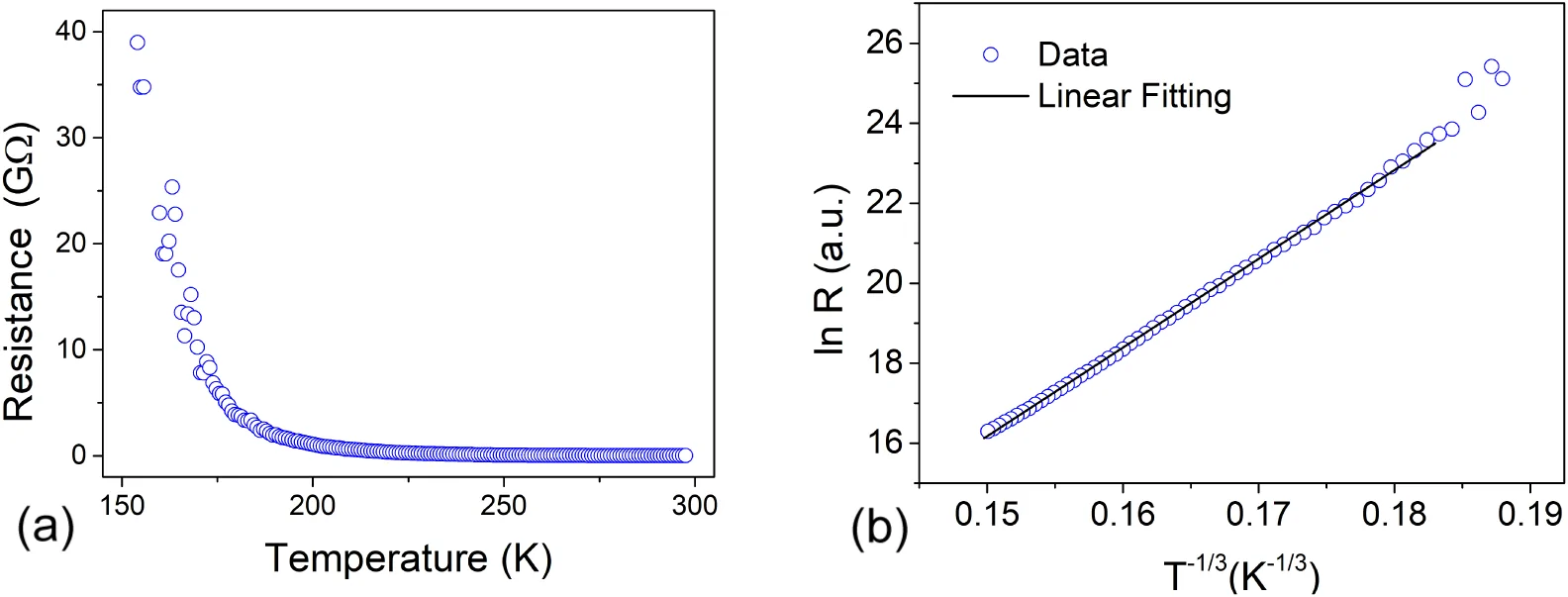
(a) Temperature-dependent resistance measurements of a few-layer MoS_2_ device; (b) fitting of the 2D VRH theory to the experimental data.

VRH is a usual carrier transport observed in semiconductors with some degree of disorder, in which the periodicity of the crystalline potential is somehow interrupted, leading to localized energy states.^29,30^ The model is based on the presence of localized energy levels around the Fermi level that trap carriers, and the transport occurs as a result of carrier hopping from an occupied to an empty state, close in energy.

In the Mott theory of VRH, the relationship between temperature and resistivity, and consequently the resistance, is given by eq 2:

2 $$\rho \left(T\right)=\rho_{0}\exp{\left(\frac{T_{0}}{T}\right)}^{1/\left(d+1\right)}$$

where ρ_0_ and *T*_0_ are constants and *d* represents the dimensionality of the system.^31^ The best fit was obtained for the exponent 1/3 (when *d* = 2), as previously observed in a MoS_2_ transistor-type device^32^ and in a device fabricated from exfoliated MoS_2_ nanoflake.^33^ This result indicates that localized states play a key role in carrier transport in our few-layer MoS_2_ device, which in turn confirms its 2D nature.

The mean hopping distance *r*_hop_ between the localized states can be derived directly from Mott’s arguments. Maximizing the expression for the optimum hopping distance,^29^ considering that in a 2D system the average separation of level energies is Δ*E* = 1/*N*π*r* ^2^, we find eq 3:

3 $$r_{hop}\left(T\right)={\left(\frac{1}{\pi \alpha NkT}\right)}^{1/3}$$

where *N* is the number of states per unit area at the Fermi level, α depends on the overlap of the wave functions, and the other symbols have their usual meanings. Relating eq 3 to the Mott and Davis expected value of *T*_0_ in two dimensions, defined by eq 4,^30^

4 $$T_{0}=\frac{3\alpha^{2}}{Nk}$$

it can be inferred that the average hopping distance in a 2D system will be calculated by eq 5:

5 $$r_{hop}\left(T\right)={\left(\frac{1}{Nk}\right)}^{1/2}{\left(\frac{1}{\pi T}\right)}^{1/3}{\left(\frac{3}{T_{0}}\right)}^{1/6}$$

Considering that the density of localized states near the Fermi level is not known in advance, its value must be determined through an experimental procedure.

To investigate the energy levels and the distribution of localized states that participate in the VRH-assisted carrier transport, thermally stimulated current (TSC) spectroscopy was performed. TSC spectroscopy, which is applicable exclusively for resistive materials,^34^ involves filling carrier traps at a low temperature and measuring the thermal emission of carriers from these traps upon warming. If there are carrier trapping centers with energy between the Fermi level and the semiconductor conduction band, for instance, the trapped carrier will be in a nonequilibrium state, and thereby, their rate of release will depend mainly on the phonon energy available in the lattice; therefore, localized states at a certain energy level begin to emit at a characteristic temperature. At first, the emission rate increases rapidly; however, as the number of filled trap states decreases with the temperature rise and there is no further injection of carriers due to the Schottky barrier, the probability of emission decreases, and the excess conductivity, in relation to dark current, exhibits maximum values.^35,36^

To perform TSC measurements, the sample was cooled to 150 K and exposed to white light to fill the traps. The lamp was then turned off, and the current was measured for a fixed bias of 10 mV while the temperature was ramped up at a rate of 0.08 Ks^–1^. TSC spectra and the dark current as functions of temperature are depicted in Figure 6 for comparison. In the inset, a TSC peak around 200 K can be observed.

**Figure 6:**
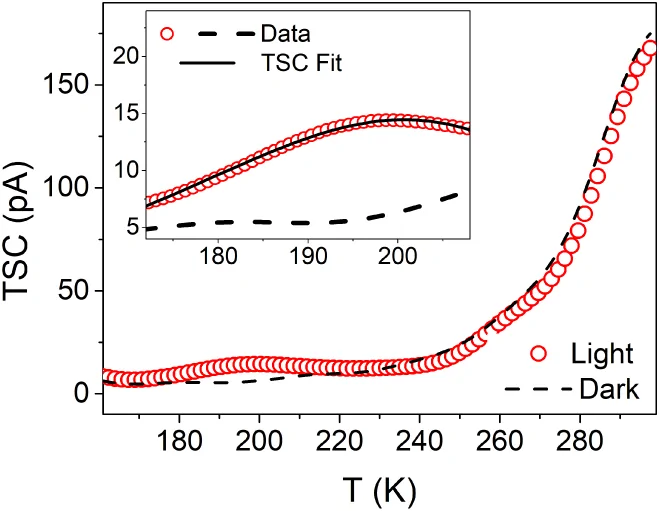
TSC data recorded from the MoS_2_ few-layer device. The dotted curve shows the dark current as a function of temperature. The fitting of the spectrum is shown in the inset.

To determine the activation energy corresponding to the peak, the TSC current data is fitted using eq 6:^36^

6 $$I\left(T\right)=I_{0}N_{C,V}\exp\left(-\frac{\Delta E}{kT}-\frac{1}{N_{T}\beta \tau }\int_{T_{TF}}^{T}N_{C,V}\exp\left(-\frac{\Delta E}{kT}dT\right)\right)$$

where *I*_0_ is related to the trap properties, *N*_*C*,*V*_ is the number of carriers in the semiconductor band, τ is the carrier lifetime, *N*_*T*_ is the density of traps, *T_TF_* is the trap filling temperature, β is the heating rate used in the experiment, and Δ*E* is the activation energy. Because few-layer MoS_2_ forms a 2D structure, the temperature dependence on *N_C,V_* has an exponent of 3/2, but this and other dimension-dependent terms in the equation are incorporated into the parameters obtained from the fit. Therefore, although some parameters have different meanings depending on the system’s dimensionality, the form of the equation and its temperature dependence remain essentially the same.^34^

The theoretical fit, also shown in the inset of Figure 6, yields an activation energy of approximately 120 meV. These localized energy states have already been linked to sulfur deficiency-related defects.^34^ It is worth noting that the TSC peak is quite broad, indicating a distribution of states around the calculated value. However, the lack of additional information about the trap properties (cross section, lifetime, etc.) makes it impossible to calculate the density of localized states from the TSC data, which is the same problem we face above, dealing with hopping analysis.

In monolayer or few-layer devices, due to the large surface area relative to its thickness, there is no significant difference between the whole material (≤4 layers) and the semiconductor-metal interface region, so a more detailed study of the interface can provide information about the entire material. At this point, it is important to remember that the contact metals were chosen precisely because they do not create additional chemical states, and the data obtained will pertain to the MoS_2_. Interface states affect most device properties and are particularly important for the capacitance behavior of Schottky contacts, which, in turn, can provide reliable data on the characteristics of the interface states. Since the depletion region of the Schottky diode is on the same order of magnitude as the thickness of few-layer MoS_2_ films (4 layers ∼49 Å), an investigation of the contribution of interface states to the capacitance can provide insights into the 2D structure of MoS_2_. In the following, we will deal with the study of the capacitance of the Schottky contacts and their properties using Schottky Capacitance Spectroscopy (SCS).

The SCS method consists of following the capacitance variation under forward bias, that is, in the conduction regime of the diode. Capacitance measurements are generally taken as a function of bias and frequency^37^ to move carriers in and out of the space charge region of the diode. Capacitance in Schottky diodes is determined by the width of the space charge region (depletion region), which is bias-dependent: as the diode is reverse-biased, the depletion region is gradually depleted of carriers as the limit of that region penetrates the semiconductor. At each voltage step, a number of carriers get out of the depletion region, yielding a variation of the total charge in the semiconductor, which is measured by the capacitance. However, when the diode is forward-biased, the depletion region border moves toward the semiconductor surface. In that case, charges trapped in interface states can be accessed once the quasi-Fermi level is located at energies near those of the states. If a low-frequency, low-amplitude sinusoidal signal is superimposed on the forward bias voltage, the interface charge can be modulated, as illustrated in Figure 7a, and an interface state capacitance can be observed.^38^

**Figure 7:**
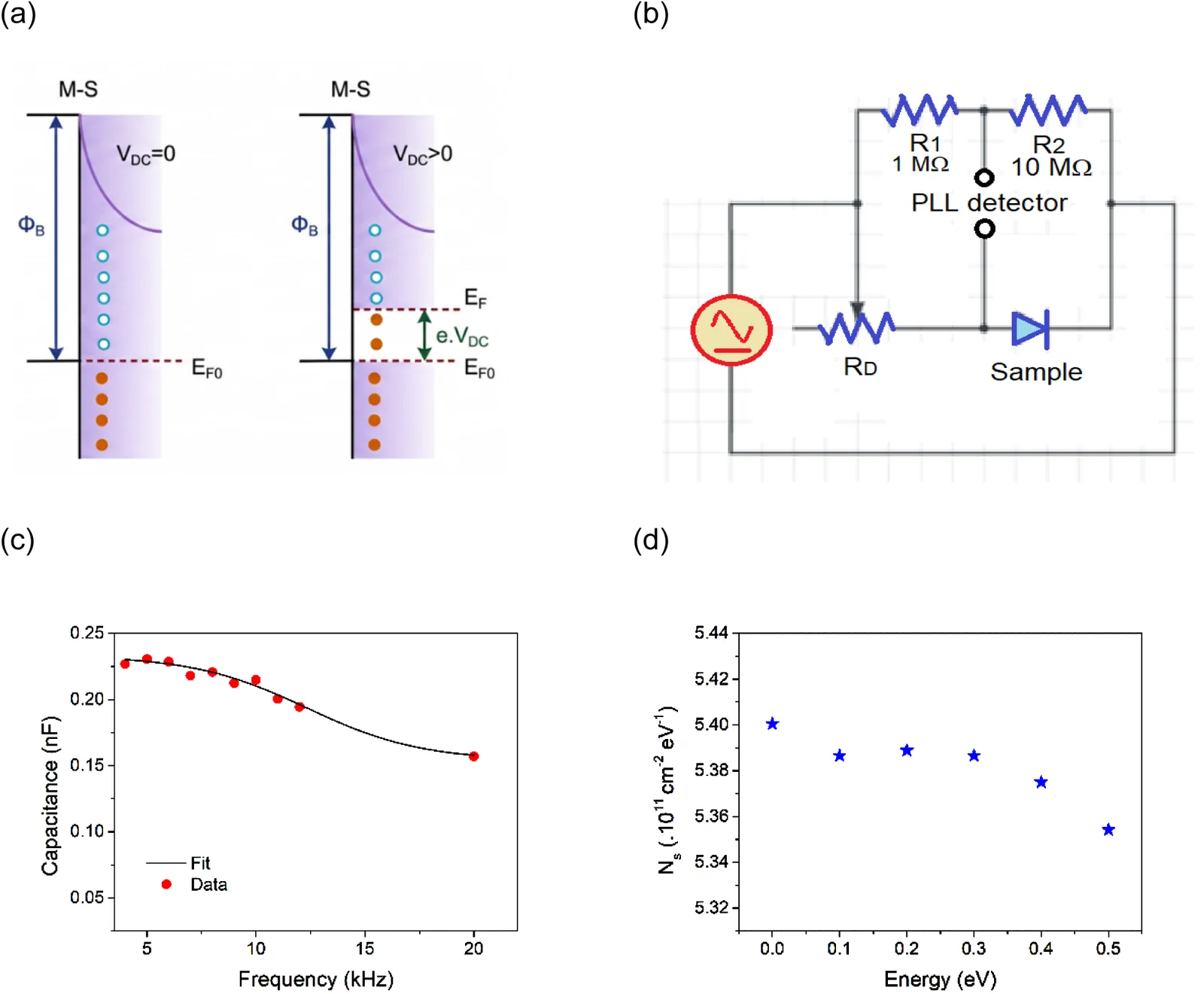
(a) Energy diagram of a forward-biased metal–semiconductor interface, where Φ_*B*_ is the barrier height, *V_DC_* is the applied DC voltage, and E_F_ is the Fermi level. (b) Electrical circuit used for SCS measurements, where *R_D_* is the variable resistance adjusted to cancel the in-phase component of the AC sinusoidal signal. (c) Interface states capacitance as a function of frequency for 0.5 V; data for forward voltages from 0 to 0.5 V are similar. (d) Interface states spectrum for In/Au-MoS_2_ by SCS.

In general, while forward bias rises gradually, there is an exponential growth of the direct current, so that it becomes difficult to measure the capacitance of the diode. Therefore, for SCS analysis, the diode was placed in a closed-cycle helium cryostat (as described previously) and incorporated into one of the arms of a bridge (Figure 7b) whose purpose is to cancel the in-phase component when a sinusoidal (AC) voltage is applied, allowing capacitance measurements.^37,38^ The bridge was supplied with different DC bias levels, from 0 to 0.5 V (related to the barrier height), to shift the Fermi level at the metal–semiconductor interface up to the semiconductor band. The small sinusoidal signal with variable frequency is superimposed on the DC signal to probe the capacitance at each DC level. The deviation from balance is measured by the phase lock loop technique using a dual-channel lock-in amplifier (AMETEK 7265). For frequencies below 4 kHz, the equivalent resistance of the circuit is very high, so the low current is not enough to charge and discharge the energy levels within the duration of one cycle, and the measurements do not reflect the total capacitance of the interface states. On the other hand, the capacitance of interface states decreases exponentially above a cutoff frequency that is inversely proportional to the state’s relaxation time, in this case around 10 kHz.

After correcting for the effect of the in-phase component, the out-phase voltage is measured, and the capacitance due to the interface states was obtained using eq 7:

7 $$C_{0}=\frac{1.21V_{L}}{2\pi fV_{AC}R_{D}}$$

where *V*_*L*_ is the out-phase voltage component, *f* is the frequency of the AC signal, *V*_*AC*_ is the intensity of the AC signal, and *R*_*D*_ is the resistance that cancels the in-phase component. Capacitance curves as a function of frequency obtained for all forward voltages were quite similar, and the presence of one plateau with a capacitance of around 0.23 nF can be observed, as shown in the example in Figure 7c.

Since the measured capacitance is due to the charge modulation of the interface states, and each curve corresponds to different positions of the Fermi level, the capacitance as a function of the direct bias voltage can provide a picture of the localized energy states situated between the Fermi level and the semiconductor band.^37,38^ The state density is calculated using eq 8:

8 $$N=\frac{C_{0}}{eS}$$

where the symbols have the same meaning mentioned earlier. For the device under study, as can be seen in Figure 7d, the picture can be described as a nearly uniform distribution, with a density of states on the order of 10^11^ cm^–2^ eV^–1^.

As pointed out by Chekir, Barret, and Vapaille,^38^ there is a correlation between the density of interface states and the ideality factor of the I–V characteristics in the way that the value of *n* increases with the density of states, especially those localized states close to the semiconductor band limits. Data from the diode initial characterization (barrier height, ideality factor) can be understood in the interface state contribution framework as found by SCS analysis. As well as in the case of localized states found in TSC data, the interface states at the MoS_2_/metal contact were previously attributed to the presence of S-vacancies due to its lower formation energy;^17^ then, since in two-dimensional structures the surface-to-volume ratio is very high, the SCS findings can be attributed to the sulfur vacancies on the samples.

Returning to the analysis of the transport process, now that we have a value for the density of states, it is possible to calculate the average hop distance using eq 5. For the temperature of 300 K, the *r*_hop_ was found in the range of 70 nm. Given that the thickness of the few-layer MoS_2_ (<49 Å) is much smaller than the average hop distance, the obtained value is consistent with the fact that it is a 2D structure.

The results of the electrical characterizations performed confirm that surface states—which, in thin layers of MoS_2_ (<49 Å), may account for the whole material—dominate charge transport and the behavior of the metal–semiconductor interface (barrier height and forward capacitance) in diode-type devices. Furthermore, as the theory of VRH was developed by assuming a constant density of states around the Fermi level, the temperature-resistance dependence results were consistent with the broadening of the TSC curve and with the data obtained from SCS, indicating a uniformly distributed density of localized energy states.

## 3 Conclusion

This work involved the growth of a few layers of MoS_2_ by the CVD method, with the two-dimensional nature of the sample confirmed by Raman and photoluminescence spectroscopy. Diode-type devices were built using different metals, which provided ohmic and Schottky contacts, allowing the study of the device’s electrical characteristics. Taking advantage of a diode configuration, thermally stimulated and Schottky capacitance spectroscopies were applied and revealed the presence of a uniform distribution of interface states. Results from both techniques are in agreement with the in-plane hopping transport also observed in a broad range of temperatures. These findings stress the role of localized energy levels around the Fermi level in controlling the interfacial chemistry of the metal–semiconductor junctions as well as the overall charge transport in few-layer MoS_2_ devices.
